# Extracellular Glutathione Decreases the Ability of *Burkholderia cenocepacia* to Penetrate into Epithelial Cells and to Induce an Inflammatory Response

**DOI:** 10.1371/journal.pone.0047550

**Published:** 2012-10-19

**Authors:** Melania D’Orazio, Francesca Pacello, Andrea Battistoni

**Affiliations:** 1 Istituto di Ricovero e Cura a Carattere Scientifico San Raffaele Pisana, Roma, Italy; 2 Department of Biology, University of Rome Tor Vergata, Roma, Italy; University of Pittsburgh, School of Medicine, United States of America

## Abstract

**Background:**

The airway surface liquid (ASL) of Cystic Fibrosis (CF) patients contains a lower concentration of reduced glutathione (GSH) with respect to healthy people. It is not known whether this defect may favor lung colonization by opportunistic pathogens.

**Principal Findings:**

We have analyzed the effects of extracellular GSH on the ability of *Burkholderia cenocepacia* to penetrate and multiply in epithelial respiratory cells. Extracellular GSH proved to be able to drastically reduce the pathogen ability to adhere and invade airway epithelial cells. This effect is correlated to a GSH-dependent increase in the number of free thiols on the surface of epithelial cells, suggestive of a change in the oxidoreductive status of membrane proteins involved in *B. cenocepacia* recognition. Moreover, treatments with GSH led to a consistent reduction of the expression of IL-8, TNF-α and IL-1β in response to *B. cenocepacia* infection.

**Conclusions and Significance:**

Extracellular GSH modulates the interaction between *B. cenocepacia* and epithelial respiratory cells and inhibits the bacterial invasion into these cells. This suggests that therapies aimed at restoring normal levels of GSH in the ASL might be beneficial to control CF lung infections.

## Introduction

Cystic Fibrosis (CF) patients typically show a marked decrease in the concentration of reduced glutathione (GSH) in their airway surface liquid (ASL) [1]. In fact, while GSH concentration in the ASL of healthy humans is in the range of 400 µM [2], in adult CF patients GSH content is approximately one third. The relationship between CFTR functionality and efficient GSH export is confirmed by the observation of comparable alterations in GSH extracellular content in the lung of CFTR knockout mice [3].

The exact functions of GSH in the lung are not known, but there are reasons to believe that the decrease of GSH in the ASL may contribute to the lung damage typical of the disease [4]. GSH is known to play very important functions related to its potent electron−donating capacity, including protection from the damaging effects of reactive oxygen species (ROS) and regulation of several cellular events, such as cell proliferation, gene expression, apoptosis and immune response [5]. High levels of GSH in the ASL could be useful to prevent inflammation and tissue damage by participating to the scavenging of the ROS spontaneously generated in this highly oxidizing environment or actively produced by neutrophils. GSH could also be involved in the control of mucus viscosity, by its ability to break disulfide bonds. These hypotheses have promoted some pilot studies aimed at analyzing the effects of GSH inhalation [6]–[9] or of the oral administration of the GSH pro−drug N−acetylcysteine (NAC) [10], [11] on the clinical status of CF patients.

Although the studies on human subjects are too preliminary to support the hypothesis that specific regimens of GSH supplementation are beneficial to CF patients [12], some recent *in vitro* studies have suggested that extracellular GSH may modulate cellular responses to insults typical of the disease. For example, GSH may control the levels of chlorinated compounds formed by the activity of myeloperoxidase, a neutrophil-released protein abundantly present in CF patients secretions [13], [14] and prevent NF-*k*B activation [14]. Other studies have shown that GSH protects cells from the toxic effects of pyocyanin (PCN) [15], [16], a major exotoxin released by *Pseudomonas aeruginosa* which significantly contributes to the pathophysiological alterations observed in the lung of CF patients chronically infected by this pathogen [17].

Interestingly, a connection between GSH levels in the ASL and resistance to bacterial infections is suggested by the observation that extracellular GSH increases to the millimolar level in the ASL of wild type mice following *P. aeruginosa* infection, whereas this response is not observed in CFTR mutant mice [4]. However, the possibility that extracellular GSH could be involved in the control of lung colonization by opportunistic pathogens has so far been poorly investigated.

In order to begin to fill this gap in knowledge, we have studied the effects of extracellular GSH on the ability of *Burkholderia cenocepacia,* an opportunistic pathogen responsible of life-threatening infections in CF patients, to penetrate into epithelial cells of respiratory origin. Our results suggest that the presence of high levels of GSH in the ASL may contribute to the control of lung infections.

## Materials and Methods

### Bacterial Strains and Growth Conditions

The *B. cenocepacia* strain LMG 16656 (*cblA+*; BCESM+), corresponding to the sequenced reference strain J2315 [18], was obtained from the Belgian Coordinate Collection of Microorganims (BCCM). The *B. cenocepacia* 6L (*cblA−*; BCESM+) is a clinical isolate from the lung sputum of a CF patient (isolate number 6 of ref [19]). The epidemic *B. cenocepacia* K56-2 [20] was a kind gift of Dr. Jorge Leitao (Instituto Superior Técnico, Lisboa, Portugal).

For most of the experiments reported in this work, bacteria grown on *Pseudomonas* Isolation Agar (PIA) plates (DifcoTM) were inoculated in chemically defined medium (CDM), containing 48 mM glucose, 7.4 mM KCl, 6 mM NaCl, 48 mM (NH_4_)_2_SO_4_, 0.5 mM MgSO_4_×7H_2_O, 60 mM MOPS, 3.8 mM K_2_HPO_4_×3H2O, 0.1% Casamino acid [21], and grown without agitation at 37°C. In addition, preliminary experiments were carried out using *B. cenocepacia* strains grown in Luria-Bertani (LB) broth (10 g/L tryptone, 5 g/L yeast extract, 10 g/L sodium chloride pH 7.1) at 37°C (with or without agitation at 150 r.p.m.).

### Host Cell Lines and Culture Conditions

The human tracheobronchial epithelial cell line 9HTEo- [22] and the cystic fibrosis human tracheobronchial epithelial cell line CFTE29o- (ΔF508/ΔF508) [23] were kindly provided by Dr. Dieter Gruenert (University of California at San Francisco, California). The cystic fibrosis bronchial epithelial cell line IB3−1 (ΔF508/W1282X) and the isogenic CFTR-complemented C38 cell line were obtained from ATCC. Cells were routinely maintained in MEM (9HTEo- and CFTE29o-) or D-MEM (IB3-1 and C38) (Euroclone) supplemented with 2 mM glutamine, 100 U/ml penicillin, 0.1 mg/ml streptomycin, and 10% heat-inactivated fetal calf serum (FCS), in a humidified 5% CO_2_ incubator, at 37°C.

All cell lines were grown in bovine serum albumin-collagen-fibronectin coated flasks and the confluent, adherent monolayers were released from the plastic surface after treatment with trypsin (C38 and IB3-1) or polyvinyl-pirrolidone-trypsin-EDTA solution (9HTEo- and CFTE29o-), collected by centrifugation at 700×g and resuspended in fresh medium. Cell viability was assessed by trypan blue exclusion.

### Adhesion and Invasion Assays

Cell monolayers were prepared by seeding 1.5×10^5^ cells/well (9HTEo- and CFTE29o-) or 1.0×10^5^ cells/well (C38 and IB3-1) epithelial cells in precoated 6-well tissue culture plates, 48 hours before infection. Cells were grown at 37°C in 5% CO_2_ and, prior to bacterial infection, were incubated for 2 hours in antibiotic-free medium, containing 2% FCS. Bacteria were grown to mid-exponential phase (OD_600_ of 0.3–0.4), harvested at 4°C, washed with phosphate-buffered saline (PBS) and then diluted in antibiotic-free medium containing 2% FCS, with or without 10 mM GSH. The buffered solution of GSH was obtained by dissolving the compound (obtained from Sigma-Aldrich) in phosphate buffered saline (PBS) and adjusting the pH to 7.4 with NaOH. The same procedure was used to prepare the solution of oxidized GSH (GSSG). The bacterial suspension was used to infect semi-confluent cell monolayers at a multiplicity of infection (MOI) of 10 bacteria/cell.

Adhesion assays were carried out by incubating infected monolayers for 2 hours at 4°C. Nonadherent bacteria were removed by rinsing four times with ice-cold PBS. Cells were treated with trypsin for 5 minutes at 37°C and lysed by the addition of 1% deoxycholic acid. Serial dilutions were performed in PBS and plated on PIA in triplicate.

Invasion assays were performed by using the ceftazidime-amikacin protection assay [24]. The infected monolayers were incubated for 3 hours at 37°C in 5% CO_2_ to allow bacterial entry. Then, infected cells were washed twice with PBS, and fresh medium containing amikacin (1 mg/ml) and ceftazidime (1 mg/ml) was added to kill extracellular bacteria. After 2 hours of incubation at 37°C, a time that we have verified is sufficient to ensure the killing of all planctonic bacteria, monolayers were washed three times with PBS, treated with trypsin for 5 minutes at 37°C and lysed by the addition of 1% deoxycholic acid. Intracellular bacteria were quantified by plating serial dilutions of the lysates on PIA plates. Invasion efficiency was calculated as the number of intracellular bacteria recovered after cell incubation in presence of antibiotics divided by the number of bacteria added to each well. Each adhesion or invasion assays included at least three independent replicates.

### Assay of Intracellular Growth

Bacterial cell suspensions of *B. cenocepacia* LMG 16656 were inoculated with or without 10 mM buffered (pH 7.4) GSH on semi-confluent monolayers as described above. After 30 minutes of incubation, the infected cells were washed with PBS, and cell culture medium containing amikacin (1 mg/ml) and ceftazidime (1 mg/ml) was added. Monolayers were then incubated at 37°C to kill extracellular bacteria. After 2 hours, the medium was replaced with antibiotic-free cell culture medium, and the intracellular bacteria were quantified over time. At each time point, the infected monolayers were lysed as described above, and the bacterial titers were determined by serial dilutions and plating.

### Primary Cell Cultures

Human bronchial CF epithelial cells (ΔF508/R553X) were obtained from Dr. Luis Galietta (Laboratory of Molecular Genetics, Istituto Giannina Gaslini) responsible of the primary cell culture facility supported by the Italian Foundation for Cystic Fibrosis Research.

Cells were grown for two passages on rat tail collagen coated culture flasks in a serum-free LHC9/RPMI 1640 (1∶1) medium with 2mM L-glutamine, 100 U/ml penicillin and 100 µg/ml streptomycin. Subsequently, to obtain polarized monolayers, the primary cells were plated at high density (5×10^5^ cells/cm^2^) on permeable supports (Snapwell, Corning, Cambridge, MA). Cells were grown under submerged conditions in DMEM/Ham’s F12 (1∶1) containing 2 mM L-glutamine, 100 U/ml penicillin and 100 µg/ml streptomycin and 2% Ultroser G (Pall, Life Science) for five days. Then, an Air-Liquid Interface (ALI) was created to promote mucociliary differentiation. Cells in ALI were maintained for 21 days at 37°C in a humidified incubator in an atmosphere containing 5% CO_2_ and then utilized for invasion experiments. Differentiation was checked by monitoring ZO-1 expression by immunofluorescence and by verifying the lack of medium leakage from the basolateral side to the apical side. The monolayers were maintained in the antibiotic-free medium for 48 hours prior to bacterial infection and then incubated with *B. cenocepacia* LMG 16656 precultivated in CDM at a MOI of 10∶1 for 3 hours. To remove mucus from the apical surface before infection, the mucociliary-differentiated cultures were washed with 3% xylitol and then were rinsed with antibiotic-free medium [25]. After three hours of infection, the monolayers were gently washed with PBS for three times and then treated with amikacin and ceftazidime (1 mg/ml each) for 2 hours to kill any remaining extracellular bacteria. Finally, intracellular bacteria were released by lysis with 0.5% Triton X-100, 50 mM EDTA, and quantified by plating serial dilutions of the lysates on PIA plates. Invasion efficiency was calculated as the number of intracellular bacteria recovered after cell incubation in presence of antibiotics divided by the number of bacteria added to each well.

### Immunofluorescence Staining and Microscopy

*B. cenocepacia* invasivity was also analyzed by microscopy on 9HTEo- cells and on primary CF cells.

Monolayers of 9HTEo- cells were prepared as described above and infected with *B. cenocepacia* LMG 16656, in absence or in presence of 10 mM GSH for 3 hours. Subsequently, cells were washed five times to remove unbound bacteria, then fixed in cold methanol for 10 min at −20°C and permeabilized with 0.1% Triton X-100. To prevent non-specific binding of antibodies, slides were incubated with 5% BSA (Biowest) in PBS for 1 hour at room temperature. Cells were then incubated with a mouse monoclonal antibody to GAPDH (Santa Cruz) and polyclonal rabbit antibody (R418) to whole lysed *B. cenocepacia* [26], diluted 1∶150 and 1∶1000 respectively in 5% BSA, for 1 hour at room temperature or overnight at +4°C. Bound antibodies were detected by incubating slides with Alexa Fluor® 488 Anti-Mouse IgG (Life Technologies) diluted 1∶1000, or with anti-rabbit IgG conjugated with Cy3 (Jackson ImmunoResearch) diluted 1∶200 for 1 hour at room temperature. Nuclei were counterstained with Hoechst and the cells were mounted and visualized under a fluorescent microscope (Olympus DeltaVision). In order to obtain a semi-quantitative evaluation of the number of intracellular bacteria with respect to epithelial cells, each field was scanned 15 times along the *z*-plane and visualized under fluorescent microscope (at least 10 fields of each specimen were acquired).

Fluorescence observations on CF primary cells were carried out using a confocal laser scanner microscope (Olympus Fluoview 1000), by exciting at 488 nm with an Argon-ion laser and at 543 nm with an Helium-Neon laser. After three hours of infection (see before), monolayers were washed three times with PBS to remove unbound bacteria and then fixed in Bouin’s solution for 10 minutes. Subsequently, the cells were washed gently another three times with PBS, and incubated with 1% BSA (Biowest) in PBS for 2 hours at room temperature. Cells were then incubated with mouse monoclonal antibody to ZO-1 (Zymed Laboratories) and polyclonal rabbit antibody (R418) to whole lysed *B. cenocepacia* [26], diluted 1∶40 and 1∶1000, respectively in 1% BSA, 0.3% Triton X-100 in PBS, overnight at +4°C. Bound antibodies were detected by incubating monolayers with anti-mouse conjugated Alexa Fluor 488 (Life Technologies), diluted 1∶1000 and anti-rabbit conjugated with Cy3 (Jackson ImmunoResearch) diluted 1∶200 in 1% BSA for 1 hour at room temperature.

### Detection of External Plasma Membrane Thiols

For detection of external plasma membrane thiols, cells were seeded in precoated 12-well tissue culture plates and grown at 37°C in 5% CO_2_. After 48 hours of incubation, monolayers were incubated for 2 hours in antibiotic-free medium containing 2% FCS. After 3 hours of incubation with or without 10 mM GSH or bacteria, the monolayers were washed and incubated in PBS alone or with 10 µM Alexa fluor 488 C_5_-maleimide (Molecular Probes) for 30 minutes at 37°C. Subsequently, the cells were washed and scraped in PBS. Labeled thiols were detected by cytofluorimetric analysis using a BD FACScalibur.

### Cytokine Analysis

The amount of cytokine mRNA was determined by quantitative real-time PCR. Total RNA from cells was isolated using the High Pure RNA isolation kit (Roche, Mannheim, Germany). Reverse transcription (RT) was performed using the High Capacity cDNA Archive kit (Applied Biosystems, Foster City, CA, USA). Real-time qPCR analyses were carried out blindly by the QuantiGene Service of the Italian Cystic Fibrosis Research Foundation, using established procedures [27].

The levels of IL-8 in supernatants (diluted 1∶20) were also measured by ELISA (R&D Systems, UK) according to the manufacturer’s instructions.

### Data Analysis

Data are expressed as mean ± standard deviation. Results were analyzed for statistical significance using the Student's *t*-test. *p* values <0.05 were considered significant.

## Results

### Effect of Extracellular GSH on *B. cenocepacia* LMG 16656 Ability to Penetrate within Epithelial Cells

It is well established that *B. cenocepacia* is able to invade epithelial cells [24], [25], [28], [29] and that different strains exhibit variable invasion ability [30]. Before carrying out experiments to evaluate the effects of extracellular GSH on the invasion of *B. cenocepacia*, we have carried out a set of experiments to identify the optimal invasion conditions. To this aim, we have compared the invasion ability of bacteria precultivated in a defined medium (CDM) or in a rich medium (LB), either using static or shaken cultures. We observed that the invasive ability of *B. cenocepacia* LMG 16656 was dramatically affected by the cultivation conditions of preinocula (**Figure S1**). In fact, not only bacteria precultivated statically were much more invasive than those precultured under shaking, but we observed that bacteria grown statically in CDM were approximately ten fold more invasive than bacteria precultivated in LB under comparable conditions. Similar results were obtained with all the strains used in this work (data not shown).

**Figure 1:**
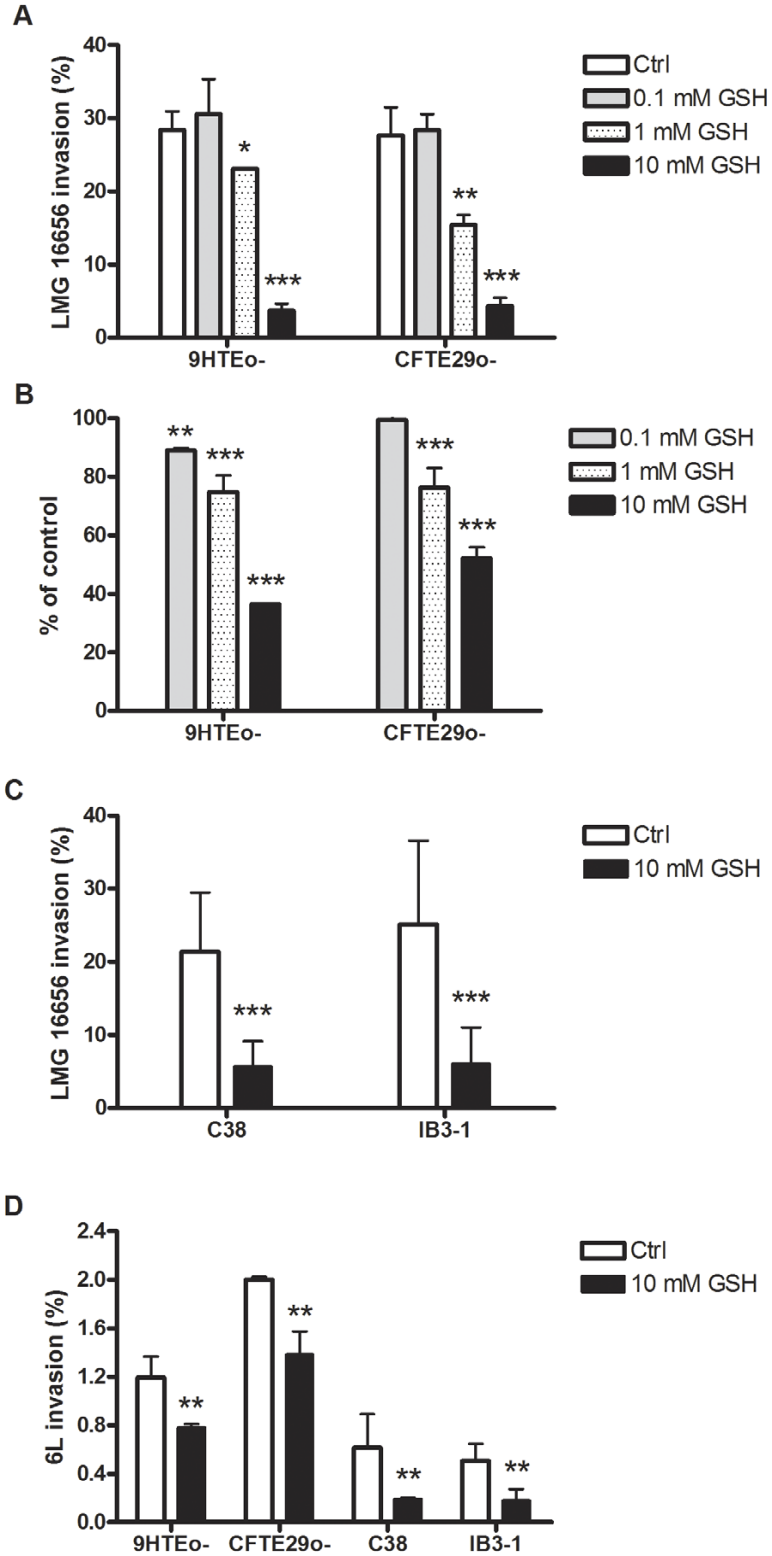
Extracellular GSH decreases *B. cenocepacia* invasion. Panel A. Invasion of 9HTEo- and CFTE29o- by *B. cenocepacia* LMG 16656 was assayed in presence of 0, 0.1, 1 and 10 mM GSH. The % of invasion indicates the ratio between the number of intracellular bacteria recovered from infected cells with respect to the bacteria added to the cell monolayer. Results represent means ± SD obtained by measuring *B. cenocepacia* LMG 16656 invasion in three independent experiments. Asterisks denote statistically significant results (* p<0.05; ** p<0.01 and *** p<0.0001, respectively). White bar: untreated cells; grey bar: cells treated with 0.1 mM GSH; dotted bar: cells treated with 1 mM GSH; black bar: cells treated with 10 mM GSH. **Panel B.** 9HTEo- and CFTE29o- were pretreated with 0, 0.1, 1 and 10 mM GSH for 3 hours at 37°C, washed to remove GSH and then used in the infection assay. Results, which are expressed as the mean ± SD of intracellular bacteria isolated from cells pretreated with GSH with respect to untreated cells, are the average of three independent experiments (**p<0.01; ***p<0.0001). Bar colors are as in panel A. **Panel C.** Invasion of C38 and IB3-1 by *B. cenocepacia* LMG 16656 in presence of 0 and 10 mM GSH. Results represent means ± SD obtained by measuring *B. cenocepacia* LMG 16656 invasion ability in three independent experiments (*** p<0.0001). White bars: control cells; black bars: cells treated with 10 mM GSH. **Panel D.** Invasion of 9HTEo-, CFTE29o-, C38 and IB3-1 by *B. cenocepacia* 6L in presence of 0 and 10 mM GSH. Results are shown as % of intracellular bacteria recovered with respect to the bacteria added to the cell monolayer. The reported values are means ± SD obtained by measuring 6L invasion ability in three independent assays (** p<0.01). White bars: control cells; black bars: cells treated with 10 mM GSH.

The effect of extracellular GSH on the ability of *B. cenocepacia* LMG 16656 to invade epithelial cells has been initially studied in 9HTEo- and CFTE29o- cells. Invasion assays were carried out either in the absence of extracellular GSH or in presence of 0.1, 1 or 10 mM GSH. **Figure 1A** shows that *B. cenocepacia* LMG 16656 has a marked capability to penetrate within these cells. In fact, at 3 hours post-infection the number of bacteria found within cells was close to the 30% of the bacteria added to the cell monolayer. Bacterial entry was not affected by 0.1 mM GSH, whereas a slight decrease in the number of intracellular bacteria was observed in presence of 1 mM GSH. Interestingly, a close to 90% reduction in the number of intracellular bacteria was observed when infections were carried out in presence of GSH 10 mM. No significant differences in bacterial entry were observed between 9HTEo- and CFTE29o- cells. Only the reduced form of glutathione was able to impair bacterial invasion, as incubations with oxidized glutathione (GSSG) up to 10 mM did not alter the number of intracellular bacteria at 3 hours post-infection (data not shown).

To evaluate if the GSH-mediated reduction of invasion efficiency could be due to effects of the antioxidant on epithelial cells or on *B. cenocepacia*, cultured cells or bacteria were pretreated with different concentration of GSH before, but not during, the infection. **Figure 1B** shows that bacterial invasion was reduced upon the pretreatment of 9HTEo- and CFTE29o- cells with 1 or 10 mM GSH. However, the reduction in bacterial invasion obtained by pretreating cells with 10 mM GSH was lower than that observed in experiments where a comparable GSH concentration was present during the invasion assay (**Figures 1A and 1B**). No changes in *B. cenocepacia* LMG 16656 invasion were obtained by preincubating bacteria with GSH (data not shown).

To ensure that the inhibition of extracellular GSH on the *B. cenocepacia* LMG 16656 invasivity was not unique to epithelial tracheal cells, we have performed invasion assay in the bronchial epithelial cells C38 and IB3-1, obtaining similar results (**Figure 1C**).

To confirm the inhibitory effect of GSH on *B. cenocepacia* LMG 16656 invasion into 9HTEo-, we have visualized and quantified by immunofluorescence microscopy the intracellular bacteria after 3 hours of infection in presence of 0 and 10 mM GSH. The results reported in **Figure** **S2** confirm that the number of intracellular bacteria visualized within 9HTEo- cells is significantly lower when infection is carried out in presence of 10 mM extracellular GSH.

### Extracellular GSH Impairs the Invasion Ability of Different *Burkholderia cenocepacia* Strains

To evaluate the effects of GSH on the invasivity of *B. cenocepacia* strains different from LMG 16656, we have repeated infection experiments with the genetically distinct isolates 6L and K56-2. As shown in **Figure 1D**, the 6L strain proved to be much less invasive than LMG 16656 (**Figure 1A**). Nonetheless, also the ability of this strain to penetrate into epithelial cells was decreased in presence of 10 mM GSH, although the effect of GSH was less marked for the 6L strain (**Figure 1D**) than for the LMG 16656 strain (**Figure 1A**). The results obtained with the K56-2 strain were similar to those obtained with the 6L strain, either for invasion efficiency or for GSH inhibition of bacterial entry (data not shown). The much higher invasion ability of the LMG 16656 strain with respect to the 6L and K56-2 strains and the more marked effect of GSH on the former strain are suggestive of differences in the modality of interaction between different *B. cenocepacia* strains and epithelial cells.

### GSH inhibits Bacterial Interaction with the Cell Surface

**Figure 2:**
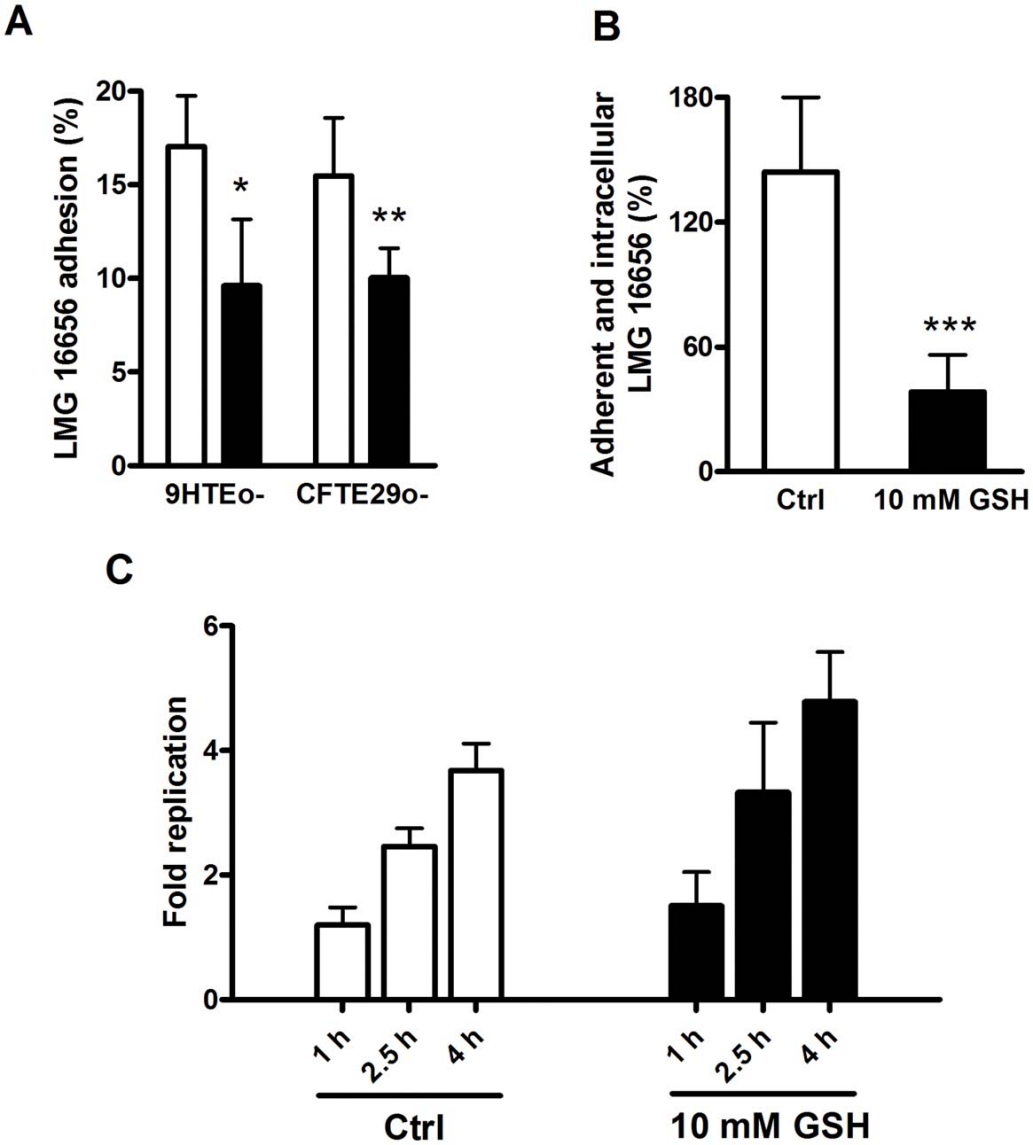
Extracellular GSH modifies *B. cenocepacia* LMG 16656 adhesion, but not intracellular replication. Panel A. Effect of extracellular GSH on *B. cenocepacia* LMG 16656 adhesion to 9HTEo- and CFTE29o- cells. Bars indicate the % of the bacteria adhering to cells with respect to the bacteria added to the cell monolayer. Results are the average ± SD of three independent experiments. White bars: control cells; black bars: cells treated with 10 mM GSH (*p<0.05; ** p<0.01). **Panel B.** Effect of 10 mM GSH on the total number (adherent + intracellular bacteria) of *B. cenocepacia* LMG 16656 recovered after 3 hours of 9HTEo- epithelial cell infection. Results are shown as % of total bacteria recovered with respect to the bacteria added to the cell monolayer. The reported values are means ± SD of three independent experiments. White bars: control cells; black bars: cells treated with 10 mM GSH (***p<0.0001). **Panel C.** *B. cenocepacia* LMG 16656 replication within 9HTEo- cells. Fold replication values were determined by dividing the intracellular bacterial load at 1, 2.5 and 4 h post-infection by that determined after 30 minutes of infection. Results are the mean ± SD of three independent experiments.

Additional experiments were carried out to examine if the reduction in the number of intracellular bacteria found in cells treated with GSH was due to an alteration in the capability of bacteria to interact with the surface of epithelial cells or in their ability to survive and replicate intracellularly. **Figure 2A** shows that GSH significantly affects the ability of *B. cenocepacia* LMG 16656 to adhere to 9HTEo- and CFTE29o- epithelial cells. We observed that extracellular GSH significantly reduces the adhesion to tracheal epithelial cells also in the case of the less invasive *B. cenocepacia* 6L strain (data not shown). Despite the described above adhesion experiments were carried out under different conditions with respect to the invasion assays (4°C vs 37°C), a role of extracellular GSH in reducing *B. cenocepacia* LMG 16656 adhesion to 9HTEo- cells is also supported by the results reported in **Figure 2B**, showing the total number of bacteria (adherent and intracellular) recovered from cells after 3 hours of incubation in presence or absence of extracellular GSH. In fact, the number of bacteria associated to 9HTEo- cells largely overwhelms that of intracellular bacteria identified through the ceftazidime-amikacin protection assay, indicating that most bacteria are likely associated to the cell surface and that extracellular GSH significantly reduces the number of bacteria interacting with cells.

In contrast, we have observed that the number of intracellular bacteria increased over time in both the cell lines and that the rate of bacterial replication within cells was not significantly affected by the presence/absence of 10 mM extracellular GSH (**Figure 2C**). These results demonstrate that extracellular GSH compromises the ability of *B. cenocepacia* to interact with respiratory cells, but has no effects on intracellular replication.

### Effect of Extracellular GSH on *B. cenocepacia* Ability to Penetrate into Differentiated Primary Cells

**Figure 3:**
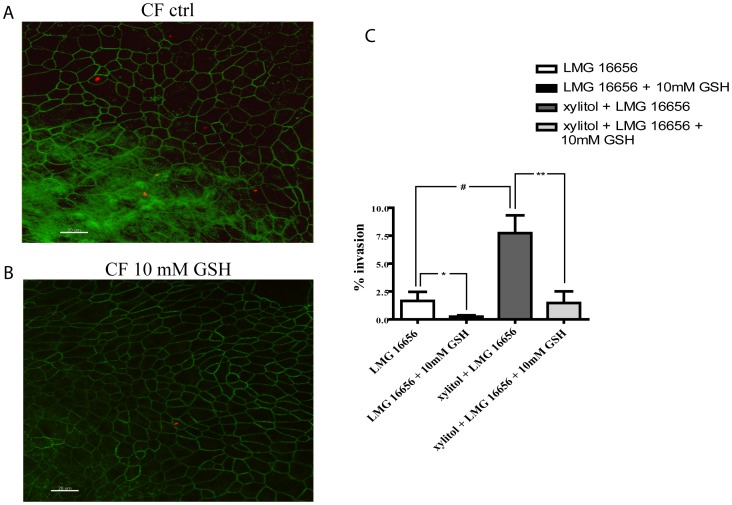
Extracellular GSH compromises the interaction of *B. cenocepacia* with mucociliary-differentiated CF bronchial epithelial cells. Panel A. MIP (Maximum Intensity Projection) from confocal system acquisition (Olympus IX 81 inverted microscope, software FV 1000) of monolayers infected with *B. cenocepacia* LMG 16656 for 3 hours, in absence (**upper**) or in presence (**bottom**) of 10 mM extracellular GSH. Cells were washed, fixed and permeabilized as described in Materials and Methods. Bacteria (red) and zona occludens (green) were detected using specific antibodies (R418 and anti-ZO1, respectively). Bar = 20 µm. **Panel B.** Invasion of mucociliary-differentiated CF bronchial epithelial cells by *B. cenocepacia* LMG 16656 in absence or in presence of GSH. Results are shown as % of intracellular bacteria recovered with respect to the bacteria added to the cell monolayers. The reported values represent the mean ± SD obtained by measuring LMG 16656 invasion ability from six individual cultures. (*p = 0.01; #p = 0.002; **p<0.05).

The effect of GSH on the invasion ability of *B. cenocepacia* LMG 16656 was also tested on mucociliary-differentiated CF bronchial primary cells (ΔF508/R553X). **Figure 3** shows that differentiated cells are more resistant to bacterial invasion with respect to immortalized non-differentiated airway epithelial cells. Nonetheless, *B. cenocepacia* LMG 16656 strain shows a remarkable ability to penetrate within such cells, which is further enhanced in cultures depleted of the apical mucus layer, according to previous observations [25]. Similarly to what observed with immortalized cells (see **Figure 1**), the ability of *B. cenocepacia* LMG 16656 to penetrate within human cells was significantly reduced by 10 mM extracellular GSH.

### GSH Alters the Redox Status of Cell Surface Thiols

**Figure 4:**
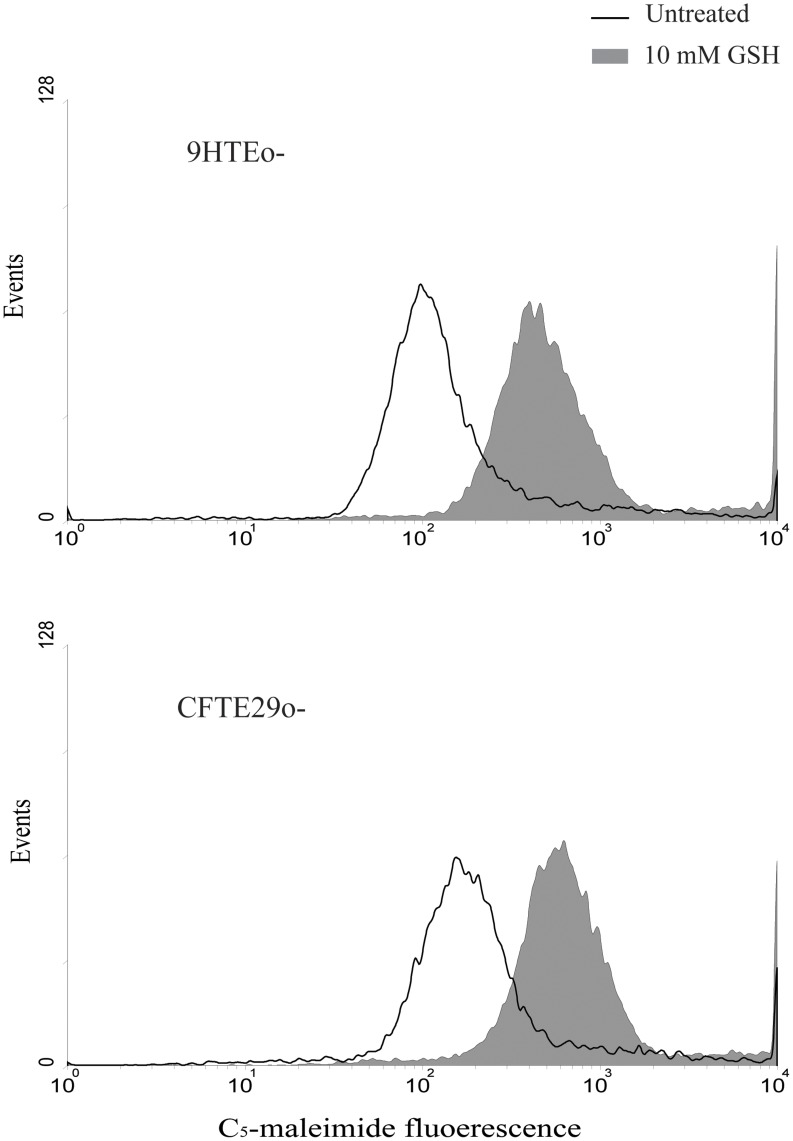
Cytofluorimetric analysis of surface thiols. After an incubation in presence or absence of 10 mM extracellular GSH, cells were treated with 10 µM Alexa fluor C_5_-maleimide to label surface free thiols and then analyzed by FACScalibur system, as described in Materials and Methods. The histograms are from a typical experiment out of three giving essentially identical results.

To test if GSH influences the redox status of thiols in proteins located on the cell membrane, 9HTEo- and CFTE29o- cells were labeled with 10 µM Alexa fluor C_5_-maleimide, a specific nonpermeable compound able to covalently bind to SH groups, after a 3 hours incubation in presence or absence of 10 mM GSH. The subsequent cytofluorimetric analysis (**Figure 4**) showed a large increase in maleimide-dependent fluorescence in cells incubated with GSH, which indicates that this treatment causes an increase in the number of surface thiols. In contrast, the presence of *B. cenocepacia* LMG 16656 did not affect the redox status of the thiols present on the cell surface, neither in presence nor in absence of extracellular GSH (data not shown). Essentially identical increase in cell-surface thiols was obtained with IB3-1 and C38 cells treated with 10 mM GSH (data not shown).

### GSH Modulates the Inflammatory Response of Epithelial Cells to *B. cenocepacia* Infection

**Figure 5:**
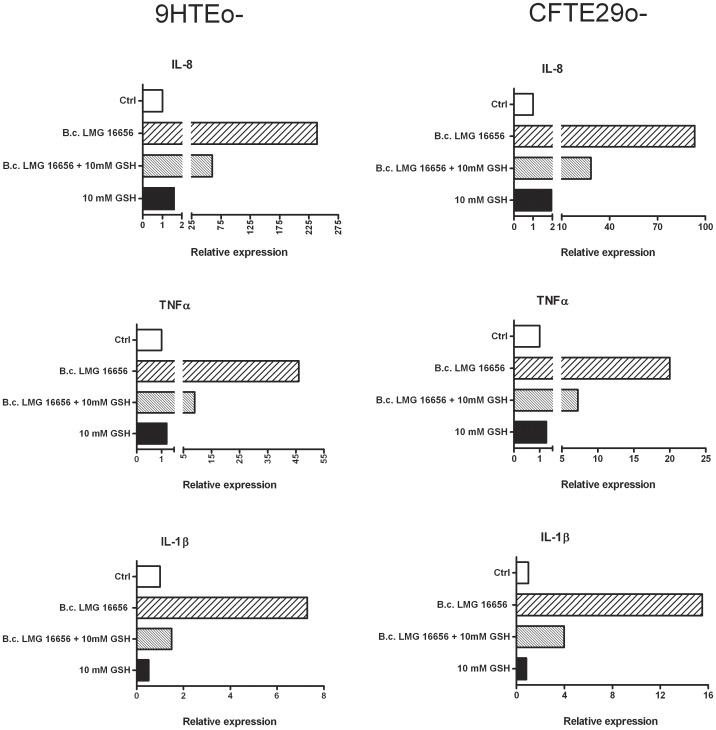
Effect of extracellular GSH on pro-inflammatory cytokines expression stimulated by *B. cenocepacia* LMG 16656. 9HTEo-, CFTE29o- cells were incubated in presence or absence of 10 mM extracellular GSH and infected with *B. cenocepacia* LMG 16656 (10 CFU/cell) in presence of 0 and 10 mM GSH for 3 hours. The expression of IL-8, TNF-α and IL-1β was analyzed by RT-PCR. Data are from a typical experiment out of three giving qualitatively similar results. Each data point is the average of three independent measures on each sample.

The ability of *B. cenocepacia* to induce an inflammatory response in respiratory epithelial cells was evaluated analyzing by quantitative real-time RT-PCR the expression of IL-8, TNF-α and IL-1β. **Figure 5** shows that the expression level of these pro-inflammatory cytokines is dramatically increased in response to *B. cenocepacia* invasion in tracheal epithelial cells 9HTEo- and CFTE29o- and that GSH significantly inhibits the *B. cenocepacia-*induced accumulation of IL-8, TNF-α and IL-1β mRNAs. Qualitatively similar results were obtained with the bronchial epithelial cells IB3-1 and C38 (**Figure S3**). In contrast, no significant induction of cytokines was observed in cells treated with heat-inactivated *B. cenocepacia*.

Production of IL-8 was measured also by ELISA. **Figure S4** shows that IL-8 can be easily detected in the supernatants from 9HTEo- cell monolayers infected with *B. cenocepacia*, but that extracellular GSH reduces secretion of this cytokine.

## Discussion

Although nearly 20 years have passed since the discovery that the concentration of GSH is reduced in the ASL of CF patients [1], it is still difficult to appreciate whether this defect has a significant role in CF lung disease. GSH plays complex functions in innate and adaptive immunity and a careful control of its homeostasis is required for a correct response to infections [31]. For example, it is well established that high levels of GSH can inhibit viral replication [32] and that low GSH enhances HIV expression via activation of NF-*k*B [33]. In contrast, little is known about the relationships between GSH status and bacterial pathogenicity, although evidences have been provided for a correlation between *Mycobacterium tuberculosis* infections and decreased levels of GSH [34]. To start an investigation on the relationships between extracellular GSH, bacterial invasion and inflammatory response in CF, we have studied the effects of GSH on the ability of *B. cenocepacia* to invade epithelial cells.

*B. cenocepacia* strains are able to penetrate within epithelial cells [24], [25], [28], [29]. We have confirmed this observation and shown that invasion ability is significantly influenced by precultivation conditions. In fact, we have found that bacteria cultivated under static conditions in a defined medium (CDM) are significantly more efficient in penetrating into respiratory cells than bacteria grown in a rich medium (LB) or under agitation. This finding resembles similar observation obtained with other bacteria, showing that the low-oxygen environment obtained by growth under static conditions enhances the ability to enter mammalian cells [35], [36]. As a matter of fact, under the conditions used in our experiments the epidemic LMG 16656 strain has an impressive capacity to penetrate into epithelial respiratory cells, whereas the 6L and K56-2 strains show a lower invasion ability.

In addition, we have found that the invasive ability of *B. cenocepacia* is drastically reduced when *in vitro* infections are carried out in presence of high levels of extracellular GSH. This effect is not due to a direct effect of GSH on the microorganism. In fact, buffered GSH does not influence neither the growth rate of *B. cenocepacia* in synthetic media nor the ability of the LMG 16656 strain, preincubated with the antioxidant, to invade epithelial cells (data not shown). Moreover, extracellular GSH, which does not permeate cellular membranes, does not affect the replication rate of intracellular bacteria.

Our observations suggest that the inhibition of *B. cenocepacia* entry is caused by effects of GSH on the membrane surface. In fact, treatments with GSH lead to a significant increase in the number of free sulfhydryls on the cell surface. The most likely explanation for such an increase in the number of exposed free thiols is that extracellular GSH regulates the oxidoreductive status of cysteine residues located on membrane proteins, favoring the reduction of exposed disulfide bonds. Although the extracellular environment is highly oxidizing, some membrane proteins, including disulfide isomerase, integrin α4, TNFα receptors (TNFRs) and the T cell surface molecule CD4 are known to expose reduced cysteines on the extracytoplasmic side of the cell [37]. Recent studies have highlighted that surface proteins containing labile disulphide bonds are much more common than previously thought [38]. The redox status of these thiols may regulate the function of these proteins. For example, the thiol status of CD4 modulates either the binding of T cells to antigen presenting cells or the entry of HIV-1 [39]. At the same time, the thiol redox status of members of the TNFR family modulates their ability to bind the cognate ligand and transduce the signal [40]. Moreover, other studies have suggested a close connection between the intracellular and the extracellular redox status, with a reducing extracellular environment regulating intracellular ROS generation [41]. Interestingly, although different *B. cenocepacia* strains bind to alternative receptors, members of the TNFR family are involved in the recognition of some specific strains [42]. Therefore, we suggest that putative receptors involved in the adhesion and penetration of *B. cenocepacia* are likely included in the proteins whose redox status is affected by extracellular GSH. The lower invasivity of the 6L and K56-2 strains with respect to LMG 16656 and the minor effect of GSH on the entry of this strain are likely due to differences in the identity of the surface proteins involved in the binding of each strain to the host cell.

Another finding that must be underlined is the identification of a clear relationship between extracellular GSH, bacterial invasion and inflammatory response. We have shown that the entry of *B. cenocepacia* within airway epithelial cells induces the expression and the release of proinflammatory cytokines and that GSH offers substantial protection against such inflammatory response. Incubation of cell monolayers with GSH does not modify the basal transcriptional levels of the genes encoding for TNF-α, IL-1β and IL-8 and no significant induction in the expression of these cytokines was observed in control experiments carried out with heat-inactivated bacteria. Rather, the action of GSH appears to be connected with the inhibition of bacterial entry within epithelial cells. In fact, the GSH-mediated reduction in the number of intracellular bacteria correlates with a lower expression of the analyzed cytokines, suggesting that the inflammatory response of epithelial cells is roughly proportional to the number of intracellular bacteria. This observation supports the hypothesis that the ability of *B. cenocepacia* to invade respiratory epithelial cells may contribute to the severe pulmonary inflammation associated to the Cepacia syndrome. Therefore, it is tempting to suggest that the low level of GSH in the ASL of CF patients may promote lung colonization by this pathogen and that GSH-based therapies could be envisioned to control *B. cenocepacia* infections.

## Supporting Information

Figure S1 **Effect of growth conditions on** ***B. cenocepacia*** **LMG 16656 invasion ability into respiratory epithelial cells.** C38 epithelial cells were infected with *B. cenocepacia* LMG 16656 precultivated under different conditions. Results are expressed as % intracellular bacteria recovered from infected cells with respect to the bacteria added to the cell monolayer, after a 3 hours of infection and 2 hours of incubation with antibiotics to kill extracellular bacteria. Values represent mean ± SD of triplicate experiments. Asterisks indicate significant differences (*p<0.05; ***p<0.0001)
(TIF)

Figure S2 **Immunolocalization of** ***B. cenocepacia*** **LMG 16656 in 9HTEo- cell culture. Panel A.** 9HTEo- cells were incubated for 3 hours with *B. cenocepacia* LMG 16656 either in the absence (ctrl, **left panel**) or in the presence of 10 mM GSH (**right panel**), then washed, fixed and permeabilized as described in Materials and Methods. Bacteria (red) and 9HTEo- cells (green) were detected using specific antibodies (R418 and anti-GAPDH, respectively). Nuclei of 9HTEo- cells were visualized by counterstaining with Hoechst 33342. Bar = 20 µm. **Panel B.** The number of intracellular *B. cenocepacia* LMG 16656 within 9HTEo- cells was determined as described in Materials and Methods. Bars represent the mean ± standard deviation of the number of intracellular bacteria divided by the epithelial cells. White bars: control cells; black bars: cells treated with 10 mM GSH.
(TIF)

Figure S3 **Effect of extracellular GSH on pro-inflammatory cytokines expression stimulated by** ***B. cenocepacia*** **LMG 16656.** C38 and IB3-1 cells were incubated in presence or absence of 10 mM extracellular GSH and infected with *B. cenocepacia* LMG 16656 (10 CFU/cell) for 3 hours. The expression of IL-8, TNF-α and IL-1β was analyzed by RT-PCR. Data are from a typical experiment out of three giving qualitatively similar results.
(TIF)

Figure S4 **Release of cytokine IL-8 in response to** ***B. cenocepacia*** **is modulated by extracellular GSH.** 9HTEo- cells were incubated for 3 hours with *B. cenocepacia* LMG 16656 (10 CFU/cell), in presence (open bar) or in absence (solid bar) of 10 mM extracellular GSH, or with medium (undetectable). Supernatants of cultures were collected and IL-8 was measured by ELISA. Values represent the mean ± SD of the mean of six individual cultures. Difference was analyzed by the Student's t-test.
(TIF)
