# Supplementary figures and images for: Extracellular Glutathione Decreases the Ability of Burkholderia cenocepacia to Penetrate into Epithelial Cells and to Induce an Inflammatory Response
Source: PLoS One. 2012 Oct 19;7(10):e47550. doi: 10.1371/journal.pone.0047550 (PMC3477146; doi:10.1371/journal.pone.0047550)

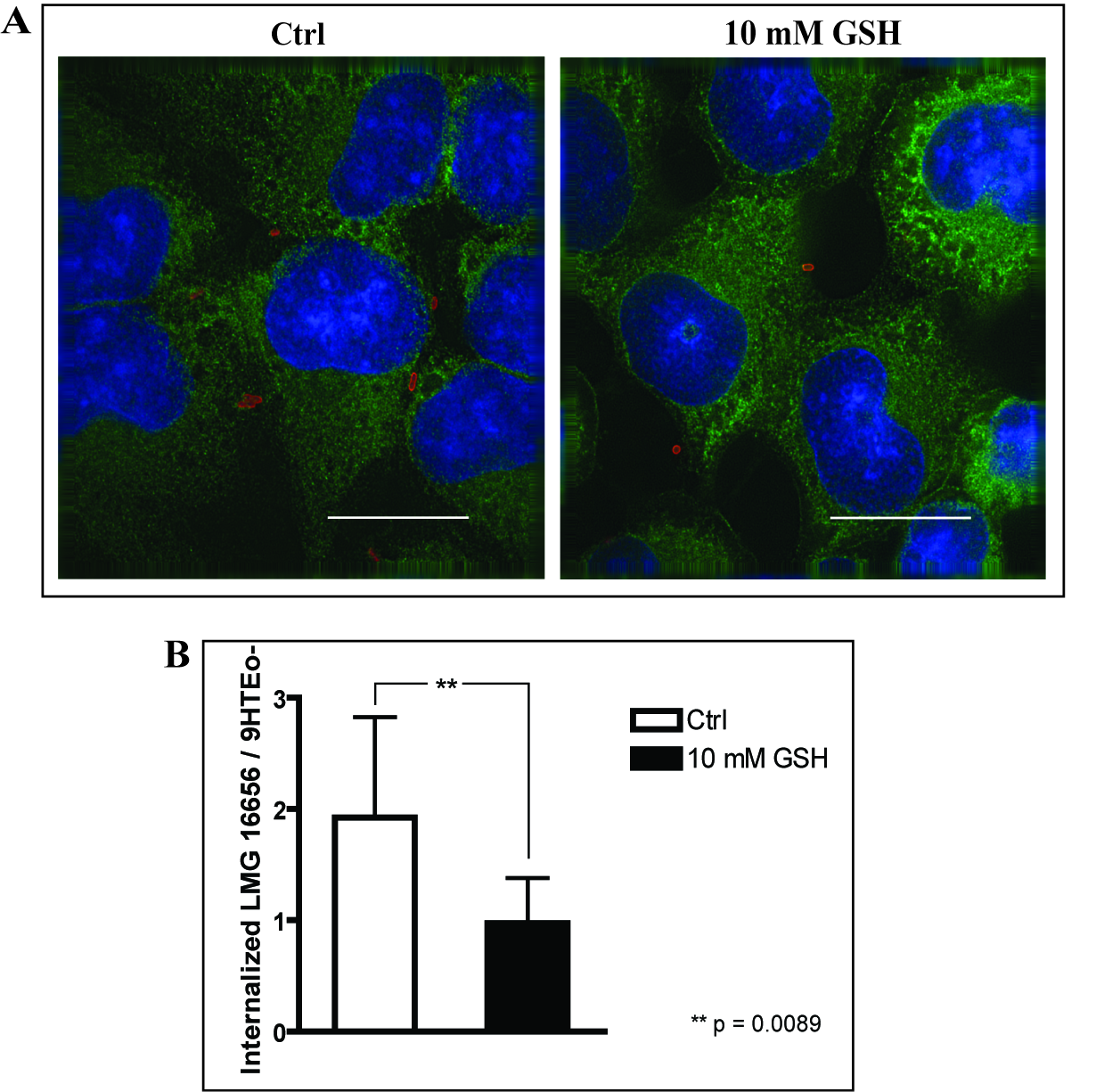

Supplement: Figure S2 — Immunolocalization of B. cenocepacia LMG 16656 in 9HTEo- cell culture. Panel A. 9HTEo- cells were incubated for 3 hours with B. cenocepacia LMG 16656 either in the absence (ctrl, left panel) or in the presence of 10 mM GSH (right panel), then washed, fixed and permeabilized as described in Materials and Methods. Bacteria (red) and 9HTEo- cells (green) were detected using specific antibodies (R418 and anti-GAPDH, respectively). Nuclei of 9HTEo- cells were visualized by counterstaining with Hoechst 33342. Bar = 20 µm. Panel B. The number of intracellular B. cenocepacia LMG 16656 within 9HTEo- cells was determined as described in Materials and Methods. Bars represent the mean ± standard deviation of the number of intracellular bacteria divided by the epithelial cells. White bars: control cells; black bars: cells treated with 10 mM GSH. (TIF) [file pone.0047550.s002.tif]
